# Supplementary material for: Eating Habits during the COVID-19 Lockdown in Italy: The Nutritional and Lifestyle Side Effects of the Pandemic
Source: Nutrients. 2021 Jun 30;13(7):2279. doi: 10.3390/nu13072279 (PMC8308479; doi:10.3390/nu13072279)
Supplement: Supplementary file 1 [file nutrients-13-02279-s001.zip › Table S1.pdf]

Table S1: The Questionnaire

Questions	Answers
**PERSONAL DATA**	
**Gender**	Male/Female
**Where do you live now?**	With my family/With flatmate or flatmates/Alone/In a specific residence/ rest home
**How many people live with you?**	People number
**Kids less than 13 are present in the family?**	Yes/No
**Level of education**	Master degree, after degree specialisation, PhD/High school/Middle school/Primary school/None of them
**Age (years)**	18 – 29/30 – 49/50 – 69/70 – 79/More than 80
**Weight**	kg
**Height**	cm
**SECTION 1- Usual eating habits**	
**Do you mainly use extra virgin olive oil to cook and flavour?**	Yes/No
**How many servings of fruit do you eat per day? 1 serving= 150 g**	None/1-2/3/>3
**How many servings of vegetables do you eat per day? 1 serving = 200 g**	None/1/2/>2
**How many servings of white bread do you eat per day? 1 serving = 50**	None/1/2/>2
**How many times do you eat whole grain cereals (e.g. pasta, rice, bread) per week?**	None/1-2/3-4 /≥5
**How many servings of red meat, hamburger, or processed meat (e.g., cured ham, salami) do you eat per week? 1 serving of red meat = 100 g; 1 serving of processed meat = 50 g**	None/1/2/>2
**How many servings of butter and/or margarine do you eat per week? 1 serving = 10 g**	None/1/2/≥3
**How many sugary drinks (e.g., coke, orange soda), including fruit juice, do you drink per week?**	None/1/2/≥3
**How many servings of legumes do you eat per week? 1 serving of fresh legumes = 150 g; 1 serving of dry legumes = 50 g**	None/1/2/≥3
**How many servings of fish or shellfish do you eat per week? 1 fish serving = 150**	None/1/2/≥3
**How many times do you eat sweets or pastries (e.g., biscuits, croissants, cakes and so on) per week?**	None/1/2/≥3
**How many servings of nuts, including peanuts, do you eat per week? 1 nut serving = 30 g**	None/1/2/≥3
**Which kinds of meat do you prefer eating?**	Mainly white meat (chicken, turkey or rabbit)/Every type of meat, including read meat (beef, pork and lamb)/None
**How many times do you eat sofrito sauce cooking tomato sauce or sautéed vegetables, per week?**	None/1/≥2
**How do you prefer drinking hot beverages such as tea or coffee?**	Adding sugar/Adding non-caloric sweetener /Without adding anything
**How many times do you eat non-whole pasta and rice per week?**	None/1/2/≥3
**How many glasses of wine do you drink per day? 1 glass = 120 ml**	Less than one glass/1-2 glasses/3-4 glasses/I do not drink wine
**How much water do you drink daily?**	Less than 1 l/Up to 1 l/Up to 1,5 l/More than 1,5 l
**SECTION 2- Changes in eating habits and lifestyle during lockdown**	

**During the lockdown how was the consumption of the following food categories? Increased, decreased or same as before?**<br>Extra virgin olive oil/Vegetables/Fruit/White bread/Whole grain cereals /Read meat/Butter and/or margarine/Sugary drinks/Legumes/Fish and shellfish/Sweets/Nuts/White meat/Sofrito sauce/Non-whole pasta and rice/Adding sugar to beverages/Wine/Water	
**During the lockdown your weight increased**	1-2 kg/3-5 kg/More than 5 kg/I do not know/My weight did not increase
**During the lockdown, how many times did you perform physical activity? (pilates, yoga, fast walking, running near your house)**	1-2 times per week/3-4 times per week/5 and more times per week /Less frequently/I did not do physical activity
**Have you changed your breakfast habits?**	I have not changed the breakfast habits/I have more time for breakfast with my family/I do not have breakfast because I eat during all morning since I am working at home
**If there are kids or young people in the family, how their eating habits have changed?**	Their eating habits have not changed/They have been involved in cooking and they are learning new things about nutrition /They are getting bored and for this reason they eat more without performing physical activity/There are no kids and young people in the family
**How habits changed during the lockdown?**<br>I need to pay attention to my spending and I should limit my purchases of expensive foods/My cooking habits did not change, I continued to cook as before/I feel I am better at cooking /I have purchased and tried new foods that I never tasted before /I cannot always find the foods I would like to eat/I ate all food I had cooked, including leftovers /I do not know how to store and consume all foods that I bought /Separate collection of waste is very difficult, I cannot do it /I eat more comfort food than before (i.e. prosecco, snacks, sweets, etc)/I eat a lot of snacks during the day /I have improved my eating habits/I need to go on a diet to lose weight/I have the perception that the elderly have difficulties with shopping and suffer from social isolation/I eat the main meals together with the rest of my family more frequently than before	For each question, choose one of the following options: Strongly disagree, Disagree, Neither agree nor disagree, Agree, Strongly agree
