# Supplementary material for: Eating Habits during the COVID-19 Lockdown in Italy: The Nutritional and Lifestyle Side Effects of the Pandemic
Source: Nutrients. 2021 Jun 30;13(7):2279. doi: 10.3390/nu13072279 (PMC8308479; doi:10.3390/nu13072279)
Supplement: Supplementary file 1 [file nutrients-13-02279-s001.zip › Table S2.pdf]

**Table S2. The sample – sociodemographic variables**

| Sociodemographic Variables | N | % |
|---|---|---|
| **Region of residence** | | |
| North | 1275 | 46.1 |
| Centre | 554 | 20 |
| South | 939 | 33.9 |
| **Gender** | | |
| Male | 1333 | 48.2 |
| Female | 1435 | 51.8 |
| **Age** | | |
| 18-29 | 403 | 14.6 |
| 30-49 | 891 | 32.2 |
| 50-69 | 911 | 32.9 |
| ≥70 | 562 | 20.3 |
| **Education level** | | |
| Low | 104 | 3.8 |
| Medium | 752 | 27.2 |
| High | 1911 | 69 |
