# Supplementary material for: Eating Habits during the COVID-19 Lockdown in Italy: The Nutritional and Lifestyle Side Effects of the Pandemic
Source: Nutrients. 2021 Jun 30;13(7):2279. doi: 10.3390/nu13072279 (PMC8308479; doi:10.3390/nu13072279)
Supplement: Supplementary file 1 [file nutrients-13-02279-s001.zip › Table S3.pdf]

Table S3. Food categories consumption before lockdown: frequency or typology

Food category		Frequency or typology of consumption			
Vegetables	*Servings/ per day*	1	2	>2	None
	%	33.9	47.2	13.5	5.3
Fruit	*Servings/ per day*	1-2	3	> 3	None
	%	68.8	16.6	5.5	9.1
Nuts	*Servings/ per week*	1	2	≥ 3	None
	%	24.9	17.7	25.5	31.9
Whole grain cereals (pasta/rice)	*Servings/ per week*	1-2	3-4	≥ 5	None
	%	24.7	22.3	27.9	25.1
White bread	*Servings/ per day*	1	2	> 2	None
	%	34.8	27.9	7.4	29.9
Non-whole pasta and rice	*Servings/ per week*	1-2	≥ 3	None	
	%	38	47.7	14.4	
Legumes	*Servings/ per week*	1	2	≥ 3	None
	%	38.1	33.5	19.3	9.1
Fish or shellfish	*Servings/ per week*	1	2	≥ 3	None
	%	41	32.4	14.6	12
Extra Virgin Olive oil to cook and flavour	*Type*	No	Yes		
	%	19.5	80.5		
Butter and margarine	*Servings/ per week*	1	2	≥ 3	None
	%	27	11.4	4.7	56.9
Sofrito sauce	*Servings/ per week*	1	≥2	None	
	%	42.5	30.1	27.4	
Red meat/hamburger/ salami/	*Servings/ per week*	1	2	> 2	None
	%	30.5	32.1	27.4	10
Favourite type of meat	*Type*	None	*Mainly white meat such as chicken, turkey and rabbit*	*Every type of meat, including read meat such as beef, pork and lamb*	
	%	7.5	37.4	55.1	
Sugary drinks	*Servings/ per week*	1	2	≥ 3	None
	%	13.7	6.3	4.9	75.1
Drinking hot beverages (coffee; tea)	*Type*	*Adding non-caloric sweetener*	*Adding sugar*	*Without adding anything*	
	%	8.9	31.4	59.7	
Sweets or pastries	*Servings/ per week*	1	2	≥ 3	None
	%	22.8	19.9	39.1	18.2
Glasses of wine	*Servings/ per day*	1-2	3-4	< 1	None
	%	15.4	4.4	39.7	40.5
