# Supplementary material for: Eating Habits during the COVID-19 Lockdown in Italy: The Nutritional and Lifestyle Side Effects of the Pandemic
Source: Nutrients. 2021 Jun 30;13(7):2279. doi: 10.3390/nu13072279 (PMC8308479; doi:10.3390/nu13072279)
Supplement: Supplementary file 1 [file nutrients-13-02279-s001.zip › Table S4.pdf]

Table S4. Cluster analysis: food consumption patterns, 4 groups

Food category	Food consumption	Total	Group 1	Group 2	Group 3	Group 4
			Healthy eaters	*Less eaters*	*Usual eaters*	*More eaters*
		100	26.8	7.5	51.4	14.3
Extra Virgin Olive oil	Decreased	2.5	2.5	8.1	0.9	5.1
	Same as before	78.6	77.6	53.7	91.7	46.7
	Increased	18.9	19.9	38.1	7.4	48.2
Fish or shellfish	Decreased	23.8	26.3	44.2	20.6	20.3
	Same as before	62.2	58.6	36.9	74.6	37.1
	Increased	14	15.1	18.9	4.8	42.5
Sweets or pastries	Decreased	12.3	12.8	61.4	4.9	12.3
	Same as before	50.8	45.9	15.8	61.7	39.1
	Increased	36.9	41.3	22.8	33.4	48.5
Nuts	Decreased	14.2	14.9	39.2	10.8	11.8
	Same as before	73.9	61.2	40.7	88.8	61.4
	Increased	12.0	23.9	20.1	0.4	26.8
White meat	Decreased	10.1	16.2	25.4	5.8	6.1
	Same as before	77.2	77.2	39.2	88.8	55.6
	Increased	12.6	6.6	35.4	5.4	38.3
Sofrito sauce	Decreased	13.8	15.3	72.4	6.6	6.2
	Same as before	76.0	74.1	19.3	87.1	69.1
	Increased	10.2	10.5	8.3	6.3	24.7
Non- whole grain cereals (pasta /rice)	Decreased	9.5	14.5	40.6	1.8	11.1
	Same as before	75.4	66.9	37.6	87.9	66.0
	Increased	15.2	18.7	21.8	10.3	22.9
Adding sugar to beverages (tea, coffe)	Decreased	7.9	6.7	4	1.3	11.8
	Same as before	89.5	90.2	48.2	96.8	83.5
	Increased	2.7	3.0	2.8	1.9	4.6
Vegetables	Decreased		9.5	11.3	9.1	6.8
	Same as before	62.5	67.2	36.2	77.8	12.5
	Increased	28.5	23.3	52.5	13.1	80.7
Fruit	Decreased	13.2	18.1	9.6	12.6	8.1
	Same as before	62.3	67.3	40.2	75.7	16.7
	Increased	24.4	14.6	50.1	11.7	75.2
White bread	Decreased	20.9	46.1	54.1	2.7	21.8
	Same as before	61.8	38	29.1	80.9	55.3
	Increased	17.2	15.9	16.8	16.4	22.9
Whole grain cereals (pasta/rice)	Decreased	9.3	8.4	27.5	5.7	14.1
	Same as before	77.6	5	56	93.7	66
	Increased	13.1	32.6	16.5	0.6	20
Red meat	Decreased	22.2	56.5	41.8	2.9	16.5
	Same as before	67.1	3	43.5	89.4	60

	Increased	10.7	8.5	14.6	7.7	23.5
Butter and margarine	Decreased	12.9	23.0	63.7	1.6	7.5
	Same as before	77.5	68.6	30.2	89.7	75.5
	Increased	9.6	8.3	6.1	8.7	17
Sugary drinks	Decreased	16.3	17.3	83.1	8.1	9.1
	Same as before	78.3	77.5	15.1	87.4	80.5
	Increased	5.3	5.2	1.8	4.5	10.4
Legumes	Decreased	7.6	9.5	19.9	5.2	6.2
	Same as before	70.3	60.3	49.5	88.2	35.3
	Increased	22.1	30.2	30.6	6.6	58.4
Wine	Decreased	12.8	7	56.9	8.5	16
	Same as before	71.2	72.1	32.3	80.9	55.2
	Increased	16.0	20.9	10.8	10.6	28.8
Water	Decreased	8.3	11.2	5.8	8.1	5.3
	Same as before	71.7	67.1	40.9	85.6	46.6
	Increased	19.9	21.7	53.4	6.3	48
