# Supplementary material for: Eating Habits during the COVID-19 Lockdown in Italy: The Nutritional and Lifestyle Side Effects of the Pandemic
Source: Nutrients. 2021 Jun 30;13(7):2279. doi: 10.3390/nu13072279 (PMC8308479; doi:10.3390/nu13072279)
Supplement: Supplementary file 1 [file nutrients-13-02279-s001.zip › Table S5.pdf]

Table S5. Clusterl analysis, the relation between the 4 groups and eating habits and lifestyle changes.
Low=Strongly disagree- Disagree; Medium= Neither agree nor disagree; High= Agree- Strongly Agree; p<0.05

**Eating habits and lifestyle changes**		**Total**	**Group 1** *Healthy eaters*	**Group 2** *Less eaters*	**Group 3** *Usual eaters*	**Group 4** *More eaters*
I cannot always find the foods I would like to eat	*Low*	54.2	47	*51.1	59.7	49.3
	Medium	17.6	17.6	14	17.3	20.1
	High	28.3	*35.4	*34.9	23	*30.5
I feel I am better at cooking	*Low*	27.1	*27.9	21.6	*29.4	19.9
	Medium	37.4	34	31.4	*41.3	33.2
	High	35.5	*38.1	*47	29.3	*46.9
I need to go on a diet to lose weight	*Low*	45.3	42.9	39.5	*50	36.1
	Medium	19.3	18.8	21.4	19.3	19.2
	High	35.4	*38.3	39.1	30.7	*44.6
I need to pay attention to my spending, and I should limit my purchases of expensive foods	*Low*	40.8	39	35.4	*43.8	36
	Medium	30.4	32.3	29	29.8	29.9
	High	28.8	28.7	*35.7	26.4	*34.1
I have purchased and tried new foods that I never tasted before	*Low*	40.1	*34.7	*38.4	47.7	*23.7
	Medium	26.7	29	25.2	26.2	24.6
	High	33.3	*36.3	*36.5	26.1	*51.7
My cooking habits did not change, I continued to cook as before	*Low*	26.7	*32.4	27.4	21.6	*33.9
	Medium	25.3	25.7	27.4	24.8	24.9
	High	48	42	45.2	*53.6	41.1
I eat more comfort food than before (i.e., prosecco, snacks, sweets, etc)	*Low*	66.1	65	*81.3	64.9	64.2
	Medium	11.3	8.2	8.2	*13.8	10.1
	High	22.6	*26.8	10.5	*21.3	*25.7
I eat a lot of snacks during the day	*Low*	64.3	62.3	70.5	65.4	60.8
	Medium	16.6	17.2	15	17	14.6
	High	19.2	20.5	14.4	17.6	*24.6
I have improved my eating habits	*Low*	45.5	40.9	31.7	*52.8	35.7
	Medium	31.8	32.3	32.3	32.2	29.4
	High	22.6	*26.8	*36	15.1	*35
I eat the main meals together with the rest of my family more frequently than before	*Low*	25.7	24.6	26	*28.3	18.2
	Medium	27.3	*26	21.1	*31.2	18.8
	High	47	*49.4	*52.9	40.5	*63
Separate collection of waste is very difficult, I cannot do it	*Low*	84.8	84	83	86.5	81.2
	Medium	8.1	8.8	9.5	7	9.8
	High	7.1	7.2	7.5	6.5	9
I do not know how to store and consume all foods that I bought	*Low*	81	81.5	83.6	80.8	79.2
	Medium	11.6	11.9	9.2	11.8	11.7
	High	7.4	6.6	7.2	7.4	9.1
I ate all food I had cooked, including leftovers	*Low*	12.2	11	13.1	12.5	12.8
	Medium	11.4	5.9	*15.1	*14.6	8.2
	High	76.4	*83.1	71.8	72.9	79
